# Exploring emotional and social competencies in undergraduate students: Perspectives from CALD and non-CALD students

**DOI:** 10.1007/s13384-022-00507-6

**Published:** 2022-04-07

**Authors:** Mong-Lin Yu, Ted Brown, Alana Hewitt, Robert Cousland, Carissa Lyons, Jamie Etherington

**Affiliations:** 1 Department of Occupational Therapy, School of Primary and Allied Health Care, Faculty of Medicine, Nursing and Health Sciences, Monash University – Peninsula Campus, Frankston, VIC 3199 Australia; 2 Student Academic Support Unit, Faculty of Medicine, Nursing and Health Sciences, Monash University – Peninsula Campus, Frankston, VIC Australia

**Keywords:** Culture, Students, Emotional intelligence, Education

## Abstract

With the internationalisation of higher education, students from culturally and linguistically diverse (CALD) backgrounds represent a significant proportion of the university student body in Australia. Research literature indicates that the unique cultural and linguistic challenges experienced by these students may adversely impact their academic performance and social integration in university settings. However, less is known about how the socio-emotional competencies of undergraduate CALD and non-CALD domestic English-speaking students compare. This cross-sectional quantitative study compares the emotional and social competencies in a cohort of CALD and non-CALD occupational therapy undergraduates. Data were collected at one Australian university from a group of 360 students enrolled in the Bachelor of Occupational Therapy (Honours) course. Participants completed the Emotional and Social Competency Inventory– University edition (ESCI-U). Multivariate regression analyses were used to compare between CALD and non-CALD students, controlling for students’ year level of study, age, and gender. Key findings from the regression analysis included the observation of indicate that non-CALD students having significant higher scores than CALD students on all the ESCI-U socio-emotional subscales, especially the Emotional Self-control, Achievement Orientation, Empathy and Teamwork from western perspectives. No difference was found between CALD and non-CALD student on two cognitive competencies- systems thinking and pattern recognition. These findings should be interpreted with caution considering CALD students may interpret the questions differently and demonstrate the competencies in different ways. Specific emotional and social competencies that need to be strengthened in students are discussed and recommendations are made to inform the preparation of evidence-based curricula.

## Introduction

Over the last decade, internationalisation has emerged as a core mission for higher education institutions across the globe. Prior to COVID-19 pandemic, the United States (US), United Kingdom (UK), China, Canada, and Australia were ranked the top five host destinations of choice for international students in 2019 (Duffin, 2020). The total number of international students in the US increased from 671,616 students in 2008 to 1,095,299 students in the 2018/2019 academic year (Institution of International Education, 2019). This made up 5.5% of total US higher education body in 2019, with students from China accounting for the largest proportion of overseas enrolments (Institution of International Education, 2019). The UK hosted approximately 460,000 international students in 2019 (The Government of United Kingdom, 2019). Data from the Australia Government Department of Education (2019) indicate that the value of international education in Australia alone increased by 22% to $32.2 billion between 2016 and 2018. The 2019 intake of 703,500 international students from 190 countries enrolled in academic programs within Australia represents a further 10% increase from 2018. Of these students, the majority are enrolled within the higher education and vocational education and training (VET) sectors. Chinese nationals comprise the largest proportion of international students at 28%, followed by India, Nepal and Brazil at 15%, 7% and 4% respectively (Australia Government Department of Education, 2019). While there has been a decline in the number of international students in 2010–2021 due to the onset of the COVID-19 pandemic, countries offering international education recognise the consequential negative economic impact and have formulated strategic plans to attract international students back to their university campuses (Greenland et al., 2021; Yilmazoglu, 2021). For example, in the US, a renewed commitment to international education was announced in 2021 to strengthen the American higher education system (U.S. Department of Education, 2021). The Australian government is also developing a new Australian strategy for international education 2021–2030 in response to the changing environment under COVID-19 (Group of Eight Australia, 2021). This indicates that the international student numbers are expected to resume gradually and increase. Therefore, it is imperative to be proactive and continue to develop quality intercultural education in preparation for the return of international students.

Health professional courses are traditionally in the top ten higher education fields that attract international students, sharing 8% of the total number of international student enrolments within Australia (IEAC, 2013). Increased student diversity is reflected in the number of international students enrolled in Australian occupational therapy programs. While many international students enrolling in Australian occupational therapy courses chose to return to their home countries at the start of the COVID-19 pandemic, most of them continued with their studies online from overseas. It is estimated that the number of overseas students enrolling in Australian occupational therapy programs varies between 5 and 40% of total student numbers. Most of this group of students originate from non-English-speaking countries and/or non-Western cultures, such as mainland China, Hong Kong, Taiwan, Vietnam, South Korea, Thailand, Malaysia, India, Pakistan, Brunei, Oman, United Arab Emirates, and Saudi Arabia (Yu et al., 2017).

There has been a perceptible shift towards universities recognising the need for institutional, curriculum and pedagogical changes that strengthen intercultural education (Jain, 2019; Mitchell & Paras, 2018). Put another way, rather than just continuing with the earlier practices of simply expecting overseas students to adjust and adapt (Arthur, 2017; Wang et al., 2015), the Australian higher education system is now more actively accommodating the needs of overseas students. Education systems are now expected to create learning environments that facilitate and uphold interculturality, respectful debate, cultural awareness and intercultural competence. In turn, this promotes academic achievement and well-being for all students through processes that develop their sense of belonging at university campuses, satisfaction with life, the reduction of anxiety while studying at university and motivating them to achieve (Beagan & Chacala, 2012).

While acknowledging the positive benefits of cultural enrichment associated with the globalisation of higher education, it is also important to recognise that the internationalisation of education has also generated increased complexities and challenges in those countries hosting overseas students. Cultural dissonance, the sense of confusion and disagreement that can be experienced by individuals in the process of adapting to a novel cultural environment, is reported to be experienced by culturally and linguistically diverse (CALD) students (Hart & Sriprakash, 2018; Lim et al, 2016). This is due to the discrepancies among CALD students’ inherent cultural values, beliefs and behaviours with pedagogy used in the host countries and impacting academic performance (Hart & Sriprakash, 2018; Lim et al, 2016). Students can come from various cultural backgrounds, present with different learning styles and language proficiency levels and can have different expectations on how teaching should be delivered, all of which can be very different from local students in the host countries (Bickel & Jenson, 2012). The important competencies for educators in those host countries are the knowledge of the cultures of both local and CALD students and the ability to manage the complex diversity of learning styles and needs within the student cohorts they engage with (Grant et al., 2014; Malau-Aduli et al., 2019). This supports the importance of intercultural education and cultural competence training in today’s higher education sector, particularly within the health profession courses which attract a high proportion of international and CALD students (Malau-Aduli et al., 2019; Richards & Doorenbos, 2016).

According to a recent Organization for Economic Cooperation and Development (OECD) report (2018), the factors that link to CALD students’ cultural dissonance and its influence on their successful adjustment to and engagement with unfamiliar educational and social environments are multidimensional. This incorporates academic (reading and writing proficiencies), social (sense of belonging), emotional (life satisfaction and levels of anxiety associated with studies) and motivational (the desire to achieve) variables (Organization for Economic Cooperation & Development, 2018). Competencies in these areas need to be closely relevant to students’ experiences in academic and workplace learning environments. For example, English language proficiency can impact communication with classmates, academic staff, and fieldwork education supervisors (Lim et al., 2016); cultural shock may result in students struggling to adapt to and meet social and academic expectations (Contreras-Aguirre & Gonzalez, 2017); and financial and cultural factors can place significant family pressure on students to succeed (Edgecombe et al., 2013).

Lim et al. (2016) refer to cultural dissonance experienced by students transferring from Asian-Pacific countries with teacher-centred education systems that do not encourage students to voice their own opinions or challenge accepted knowledge and conventions. This can lead to students experiencing difficulties adjusting to Western pedagogical systems in which they are required to contribute to group discussions, think critically, learn independently and engage with supervisors (Wang et al., 2015). The impact of culture and language as determining factors in the academic pathways of overseas health professional students, where difficulties adapting to unfamiliar academic and social environments are encountered, is widely reflected in the medical, nursing and allied health literature (Crawford & Candlin, 2013; Gilligan & Outram, 2012; Jeong et al., 2011; Lee et al., 2019; Mitchell et al., 2017).

Research in the nursing and occupational therapy arenas suggests that international students for whom English is a second language frequently encounter communication, language and cultural barriers that impact their performance in both the university classroom and field placement (also referred to as practice education) environments (Edgecombe et al., 2013; Lim et al., 2016). Challenges experienced include issues forging effective social interactions in the local community, difficulties understanding local accents, the use of colloquialisms and metaphors, and the application of Confucian philosophical ideas that educators are highly respected and should not be challenged in an unfamiliar pedagogical environment that values discussion, debate and questioning (Abu-Arab & Parry, 2015; Li et al., 2017; Lim et al., 2016; Young, 2017). These are shown to create anxiety and stress in students, as well as feelings of low self-confidence and loneliness with adverse consequences for academic performance and students’ capacity to learn core practice skills and develop professional competencies (Edgecombe et al., 2013).

For CALD students enrolled in health professional courses, practice education placements (also referred to as fieldwork, internships, and clinical practicums) can be especially challenging, as students encounter new and unfamiliar environments, learning expectations (e.g. communication with clients and supervisors) and modes and nuances of professional and social interactions that can create obstacles for them (Attrill et al., 2015). The evidence is that for CALD students embarking on practice education placements, the integration process is particularly stressful in the early stages due to linguistic, social and cultural differences (Mikkonen et al., 2016). The academic resilience of Asian students as a result of their experiences of striving to succeed and get ahead within highly competitive Confucian education systems has been reported; however, this can act as a barrier to engaging in the collegiate working practices expected within Western pedagogies (Li, 2017).

Lim et al.’s (2016) study of students from Hong Kong, Malaysia and Singapore enrolled in an Australian occupational therapy program describes how international students often felt less competent than domestic students in terms of language proficiency, confidence and ability to express themselves. Expectations to be more assertive, show initiative and participate in activities were particularly stressful for students who were acutely aware that failure to demonstrate these behaviours could result in course failure (Lim et al., 2016). This serves to highlight the importance of initiatives and supportive supervision for CALD students that mitigate stress, facilitate the learning process in the classroom and also promote and ensure positive social and interpersonal interactions with colleagues and clients when undertaking practice education placements. Examples of targeted programs include pre-placement induction programs, cultural and language courses allowing additional time for CALD students to settle, and faculty support for both students and academic/fieldwork staff (Mikkonen et al., 2016).

While the ‘lived’ experiences of international students are relatively well understood (Lalor et al., 2019; Lim et al., 2016), less is known about how the self-reported socio-emotional competencies of native English-speaking and CALD students enrolled in health professional education programs compare. It is recognised that a wide range of cultural factors influences socio-emotional competencies in higher education including cultural expectations, language, race, class and gender (Hecht & Chin, 2015). Emotional intelligence (EI) and social competency are learned capabilities that contribute to success in all facets of life. For students enrolled in health training programs, emotional and social intelligence represent an array of non-cognitive skills and capacities such as professionalism, empathy and integrity that influence a person’s ability to cope with environmental demands and pressures (Talarico et al., 2013). Emotional intelligence includes self-awareness of emotions which facilitates deeper understanding of the client’s/patient’s feelings and concerns, and assists in establishing a respectful therapeutic relationship between patient and practitioner (Di Lorenzo et al., 2019).

However, what are perceived as being socio-emotional competencies can vary across different cultures. Asian culture, which emphasises social order, conformity to group cultural norms, and hierarchy tends to encourage emotional suppression, whereas Western culture emphasises egalitarianism, favours emotional expression and direct communication, and promotes individualism (Min et al., 2018). These factors influence how students manage personal and professional relationships. Hence, CALD students may report a different set of socio-emotion competencies compared to native English-speaking students. However, how important is it to understand CALD students’ self-reported socio-emotional competencies in the context of the existing expectations within the environment where they engage in learning activities and to explore the possibility for promoting intercultural education?

The aim of this study therefore was to explore differences in the self-reported levels of emotional and social competencies in CALD and non-CALD occupational therapy undergraduate students. This study is significant in that it is the first of its kind to compare the self-reported socio-emotion competencies of CALD and non-CALD students enrolled in an undergraduate health professional course. This makes a direct and needed contribution to the relevant body of empirical evidence as well as contributing to inclusive and culturally sensitive health professional higher education that considers students’ well-being. Being the first study that specifically examines the self-reported socio-emotion competencies of CALD students within the western education context will add knowledge about the challenges that CALD students may encounter and guide the development and implementation of informed, evidence-based educational support. The findings will establish a baseline of occupational therapy students’ emotional and social skills that will inform the preparation of intercultural-focussed, evidence-based undergraduate curricula, learning and teaching methods used and help identify key emotional and social competencies that need to be strengthened in CALD and non-CALD occupational therapy undergraduate students.

## Methods

### Design

This cross-sessional study used a survey design to collect data and recruited participants using convenience sampling. A cut-off of *p* < 0.05 was used to determine whether findings were statistically significant.

### Participants

A minimum sample size of 129 participants is required based on multiple regression analysis with a medium effect of ρ = 0.15, alpha set at 0.05, and power set at 0.95. Using a convenience sampling method, a total of 455 first-, second-, third- and fourth-year students, enrolled in a four-year accredited Bachelor of Occupational Therapy (Honours) course at one university in Australia were invited to participate in this study. Inclusion criteria for the study were: i) being enrolled in an accredited undergraduate occupational therapy course at XXXX University; and ii) providing consent to take part in the study. A total of 360 students (79% response rate) consented to participate in the study.

### Instrumentation

A paper-based survey that contained demographic questions regarding year level, type of enrolment (full time or part time), age, gender and status (domestic or international) and the *Emotional and Social Competency Inventory – University edition* (ESCI-U) (Korn Ferry, 2017) was used in this study.

The ESCI-U (Korn Ferry, 2017) measures participants’ intrapersonal recognition and management of their own and others’ emotions and how these influence interpersonal interactions with other people (Boyatzis, 2016). Likewise, the ESCI-U generates outcomes for assessment and development purposes and improved understanding of the role of emotional and social intelligence in learning and performance. The ESCI-U is a standardised 70 item self-report scale that measures 12 emotional and social competencies of undergraduate and graduate university students. These competencies are categorised into four broad areas: Self-Awareness, Social Awareness, Self-Management and Relationship Management. The ESCI-U also measures two cognitive competencies, Systems Thinking and Pattern Recognition. Each item in the ESCI-U was scored using a 5-point Likert scale (1 = Never, 2 = rarely, 3 = sometimes, 4 = often, 5 = consistently) representative of the frequency range in which the student self-rates a behaviour. A competency score is calculated by taking the mean of the total score of all items clustered under each competency, and a higher competency score on the scale demonstrates higher social and emotional competence (Korn Ferry, 2017).

The ESCI-U is reported to have acceptable to good internal consistencies (Cronbach’s alpha = 0.71 to 0.86) among the competencies. Appropriate convergent validity has been reported (composite reliabilities = 0.80 to 0.90; and the average variance extracted = 0.52 to 0.69). As well, confirmatory factor analyses have demonstrated appropriate model fit of each scale ($\chi 2(1398, 1921)$= 6,606; RMSEA = 0.04; PCLOSE = 1.00; CFI = 0.87; PCFI = 0.81; GFI = 0.86; SRMR = 0.47) (Boyatzis, 2016).

### Data collection

Students were invited to participate in the study in March 2019. Students were provided the survey forms with the study information by a non-teaching staff at the end of a lecture in the university where the research was conducted. Students participated on a voluntary basis and gave informed consent by self-completing and returning the survey to a survey return box located in the study university during March and April 2019.

### Data management and analysis

The data from the returned survey forms with no missing information were entered onto the Stata 14.2 (StataCorp LLC, College Station, Texas) for analysis. Chi-square was used to compare the demographic between the CALD and non-CALD student participant groups. Each of the 12 ESCI-U competencies were utilised as the dependent variables. Multivariate regression analyses were used to compare between CALD students and non-CALD students, controlling for students’ year level of study, age, and gender. Regression analyses having a p-value smaller than 0.05 are considered to be statistically significant.

### Procedures

Ethics approval for the project was obtained from the Monash University Human Research Ethics Committee (Project Number: 17590). Student participants were informed of the purpose of the study, the voluntary nature of their participation and the procedures to ensure their anonymity in all published outputs. Student participants were asked to complete a questionnaire containing demographic questions and the ESCI-U instrument. The questionnaire took approximately 20 min to complete, and consent on the part of the student participants was implied by its completion and return. No data were identifiable.

## Results

### Demographic results

The full demographic findings for the 360 student participants are reported in Table 1. Given that occupational therapy has been traditionally a female-dominant profession, the salient features of the sample were the predominance of female students under the age of 24 years, undertaking their first or second year of study and enrolled on a full-time basis.

**Table 1:** Demographic data (*n* = 360)

	All students (*n* = 360, 100%)		CALD students (*n* = 150, 41.67%)		Non-CALD students (*n* = 210, 51.33%)		Chi-square Comparison^a^	
Demographic variable	*n*	%	*n*	%	*n*	%	$\chi 2$(*df*)	*p*
Study year level							3.46 (2)	0.32
Year 1	137	38.06	56	37.33	81	38.57		
Year 2	99	27.50	48	32.00	51	24.29		
Year 3	87	24.17	34	22.67	53	25.24		
Year 4	37	10.28	12	8.00	25	11.90		
Enrolment							0,93 (1)	0.33
Full time	352	97.78	148	98.67	204	97.14		
Part time	8	2.22	2	1.33	6	2.86		
Age							3.52 (2)	0.17
17–19 years old	152	42.22	66	44.00	86	40.95		
20–24 years old	177	49.17	76	50.67	101	48.10		
25 + years old	31	8.61	8	5.33	23	10.95		
Gender							3.03 (1)	0.08
Female	278	77.22	109	72.67	169	80.48		
Male	82	22.78	41	27.33	41	19.52		
International students							282.93 (1)	0.001***
Yes	138	38.33	134	89.33	4	1.90		
No	222	61.67	16	10.67	206	98.10		

*a Demographic comparison between CALD and Non-CALD students using Chi-square; n* number of participants, *CALD* Culturally and linguistically diverse, $\chi 2$ Chi-square, *df* degree of freedom, *p* p-value (*p < 0.05; **p < 0.01; ***p < 0.001)

### Emotional and social competency scores

The full mean scores are provided in Table 2. Higher scores across all ESCI-U subscales were noted for non-CALD students compared to their CALD student peers. On measures of emotional awareness and management, both groups of students recorded their highest scores on the Achievement Orientation (CALD $\overline{x }$ = 3.81, SE = 0.04; non-CALD $\overline{x }$ = 4.02, SE = 0.04), Emotional Self-Awareness (CALD $\overline{x }$ = 3.67, SE = 0.42; non-CALD $\overline{x }$ = 3.85, SE = 0.05) and Positive Outlook (CALD $\overline{x }$ = 3.66, SE = 0.05; non-CALD $\overline{x }$ = 3.85, SE = 0.04) subscales, which correspond to occasional demonstration of emotional competencies.

**Table 2:** Emotional and Social Competencies Inventory comparative mean scores (*n* = 360)

ESCI subscales	Mean^a^ (SE) CALD students	Mean^a^ (SE) Non-CALD students	B	SE	*p*	95% CI	R^2^
Self-Awareness							
Emotional Self-Awareness	3.67 (0.42)	3.85 (0.05)	− 0.17	0.07	0.016*	− 0.31, − 0.03	0.03
Self-Management							
Achievement Orientation	3.81 (0.04)	4.02 (0.04)	− 0.20	0.06	0.002**	− 0.33, − 0.07	0.04
Adaptability	3.56 (0.04)	3.68 (0.04)	− 0.12	0.06	0.037*	− 0.24, − 0.01	0.04
Emotional Self-control	3.52 (0.38)	3.78 (0.05)	− 0.27	0.07	0.001***	− 0.41, − 0.14	0.05
Positive Outlook	3.66 (0.05)	3.85 (0.04)	− 0.20	0.07	0.002**	− 0.34, − 0.07	0.05
Social Awareness							
Empathy	3.87 (0.04)	4.11 (0.04)	− 0.24	0.06	0.001***	− 0.36, − 0.12	0.05
Organisational Awareness	3.76 (0.04)	4.07 (0.04)	− 0.32	0.06	0.001***	− 0.44, − 0.20	0.08
Relationship Management							
Conflict Management	3.46 (0.04)	3.74 (0.04)	− 0.29	0.06	0.001***	− 0.40, − 0.17	0.07
Coach and Monitor	3.37 (0.04)	3.62 (0.05)	− 0.25	0.07	0.001***	− 0.39, − 0.11	0.05
Influence	3.53 (0.04)	3.68 (0.04)	− 0.18	0.06	0.004**	− 0.30, − 0.06	0.06
Inspirational Leadership	3.37 (0.04)	3.63 (0.05)	− 0.27	0.07	0.001p***	− 0.30, − 0.14	0.07
Teamwork	3.75 (0.04)	4.27 (0.04)	− 0.53	0.06	0.001***	− 0.64, − 0.41	0.21
Cognitive Competencies							
Systems Thinking	3.50 (0.04)	3.51 (0.04)	− 0.01	0.06	0.82	− 0.13, 0.11	0.02
Pattern Recognition	3.51 (0.04)	3.57 (0.05)	− 0.08	0.07	0.20	− 0.21, 0.04	0.05

a Mean score: 1 = never, 2 = rarely, 3 = sometimes, 4 = often, 5 = consistently; *ESCI* Emotional and Social Competencies Inventory, *CALD* culturally and linguistically diverse; * *p* < .05; ** *p* < .01; *** *p* < .001. ^a^Regression results controlled for students’ year level of study, age and gender

In relation to social awareness and relationship management, non-CALD students performed best on the Teamwork ($\overline{x}$ = 4.27, SE = 0.04), Empathy ($\overline{x}$= 4.11, SE = 0.04) and Organisational Awareness ($\overline{x}$ = 4.07, SE = 0.04) subscales, with scores indicating consistent demonstration of competencies. CALD students recorded their highest scores in the same domains (Empathy $\overline{x}$ = 3.87, SE = 0.04; Organisational Awareness $\overline{x}$ = 3.76, SE = 0.04; Teamwork $\overline{x}$ = 3.75, SE = 0.04); however, their lower scores were indicative of less consistent demonstration of social competencies.

### Regression analysis results

Regression analysis revealed statistically significant differences in CALD and non-CALD student scores across all ESCI subscales, except for the two cognitive competencies domain, after controlling for students’ year level of study, age, and gender. On measures of competencies in social awareness and relationship management, key findings were observations at the *p* < 0.001 level of statistically significant differences. In that, CALD students’ scores on the Empathy (β = − 0.24, *p* < 0.001), Organisational Awareness (β = − 0.32, *p* < 0.001), Teamwork (β = − 0.53, *p* < 0.001), Conflict Management (β = − 0.32, *p* < 0.001), Coach and Monitor (β = − 0.25, *p* < 0.001) and Inspirational Leadership (β = − 0.27, *p* < 0.001) subscales were significantly lower than non-CALD students. Analysis of students’ comparative scores in emotional awareness and management revealed that CALD students’ scores on the subscales measuring Emotional self-control (β = − 0.27, *p* < 0.001), Achievement Orientation (β = − 0.20, *p* < 0.01), Positive Outlook (β = − 0.20, *p* < 0.01) and Emotional Self-Awareness (β = − 0.17, *p* < 0.016) were significantly lower than non-CALD students. No difference was found for the two cognitive competencies, those being systems thinking and pattern recognition (see Table 2 for full regression statistics).

## Discussion

Exploration of the regression analysis results revealed statistically significant differences between CALD and non-CALD students' scores across all emotional and social competencies ESCI-U subscales, except the cognitive competencies domain. This indicates that international and domestic students report similar cognitive abilities and suggests the differences observed on measures of emotional and social competencies are accounted for by environmental factors. The most notable findings relate to the results observed across the emotional self-management, social awareness and relationship management subscales, as they provide further evidence of the cultural and linguistic challenges faced by CALD students as reported in the empirical literature.

It is important to note however that both CALD and non-CALD students self-reported scores across all subscales that fell in the mid-score range, which equated to occasional demonstration of the competencies measured by the ESCI-U. This reflects the composition of the current sample in which first- and second-year students under the age of 24 predominated. As younger students, it might be expected that their social and emotional competencies are still at a formative stage in contrast to older students completing their final years of study whose social and emotional capabilities are more developed and nuanced as a result of their greater experience and maturity (Rai, 2017). For non-CALD students, their scores approach a level of frequent demonstration of competencies in two areas only: Teamwork and Empathy, both components of social capability. CALD students reported their highest scores in Empathy and Achievement Orientation, which represents an encouraging finding for educators as it is consistent with levels of motivation and a desire to succeed that has previously been observed in CALD students enrolled in health science programs (Edgecombe et al., 2013). This indicated the emergence of a baseline of empathic skills in CALD students that can be built upon as students’ theoretical and practical discipline-specific knowledge progresses in the later years of study. Other than having different strengths, CALD and non-CALD students also demonstrate difference in each individual emotional and social competence.

### Emotional competencies

#### Emotional self-awareness and self-control

Awareness of one’s own and others’ emotions to enable control over disruptive feelings, impulses and thoughts, and to maintain effectiveness under stressful conditions are desirable professional behaviours in healthcare professionals (Beagan, 2015). Studies have demonstrated that the traits of emotional intelligence such as self-expression and the emotional management of others are also associated with positive professional behaviours in academic settings (Brown et al., 2016). Therefore, establishing significant differences in self-reported CALD and non-CALD students’ emotional competencies represents an important and challenging finding for educators and institutions intending to promote intercultural education development. This is particularly in the context of research that suggests CALD undergraduates’ interactions with academic staff and domestic students are often characterised by negative emotions, such as feelings of uncertainty, awkwardness and embarrassment (God & Hongzhi, 2019; Lalor et al., 2019).

Studies from the nursing arena have established that CALD international students’ difficulties are further compounded by clinical educators’ perception of them as ‘needful’ students in terms of support required and CALD students’ experiences of unmet needs and expectations (Abu-Arab & Parry, 2015; Edgecombe et al., 2013). The low scores reported by CALD students in the current study across measures of emotional self-awareness and self-control align with previous research evidence. Studies indicate that CALD students’ emotional self-esteem can be negatively impacted by their perception that domestic students represent an ‘ideal’ and thus possess desirable attributes such as the ability to fully express themselves and proficiency in English language skills that generate confidence in their academic endeavours and social interactions (Lim et al., 2016). These highlight the urgency and necessity to promote the development of intercultural education beyond the university’s academic teaching environment to practice education in clinical settings for health professional courses.

The statistically significant differences observed on these key aspects of emotional intelligence may also reflect cultural, financial and linguistic barriers which carry judgmental and affective consequences for individuals in cross-cultural situations (Edgecombe et al., 2013; Mak et al., 2014). A distinct cultural factor that has been shown to influence emotional self-awareness and impact CALD students’ integration in the academic environments of host countries is a lack of familiarity with Western-style pedagogies (Hecht & Chin, 2015). CALD students from overseas are often financially dependent on their family and under financial stress needing to pay high tuition fees plus living and accommodation costs. This stress and sense of financial dependence can have a negative impact on CALD students’ from overseas health and emotional well-being (Banjong, 2015). Domestic students are more likely to have experience with typical teaching formats that emphasise the need to learn independently, contribute to group discussions and apply critical and reflective learning, which combine to facilitate easier navigation through the university system, including practice education, for domestic students compared to their international counterparts (Wang et al., 2015).

CALD students originating from Asian societies with strong rules and expectations regarding cultural conformity and the expression of emotions are likely to be unfamiliar with new methods of learning, which are allied to linguistic challenges and can restrict their capacity to meet educators’ expectations and course requirements (Lim et al., 2016; Young, 2017). In the context of emotional capabilities, Asian societies instil a broader perspective that emphasise the importance of getting along with others and keeping emotions in check for the greater good (Young, 2017). The emphasis on self-criticism in Asian culture can also impact students’ perspectives, resulting in their being harsh on themselves when self-evaluating their social-emotional competencies (Boyraz et al., 2020).

This contrasts to domestic students who are brought up in a culture where the prevailing norm is a belief of self-enhancement over self-criticism, that the self is an autonomous, self-sufficient entity, the voicing of opinions is valued and encouraged, and beliefs and emotions are markers of one’s identity (Boyraz et al., 2020; Hecht & Chin, 2015). International students arriving from societies that adhere to social order and longer-term perspectives can therefore experience significant challenges and additional stress when transitioning to academic environments in host countries with different cultural norms, values and roles (Chang, 2015; Young, 2017).

#### Achievement orientation

Achievement orientation refers to students’ capabilities in striving to meet or exceed standards of excellence, looking for ways to do things better, setting challenging goals and taking calculated risks. Non-CALD students reported approaching frequent demonstration of these attributes; however, CALD students reported a significantly lower score. In the context of studies that characterise international students undertaking higher education in host countries as being of high intellectual ability and greater than average motivation (Lillyman & Bennett, 2014), it is possible to surmise that personal and situational factors are inhibiting CALD students’ capacities in this area. The findings in the current study link to previous research reporting significant differences on measures of self-perception and pressure to perform in international and domestic health professional students. This confirms that CALD students are subject to greater stresses derived from parental expectations and their experiences of highly competitive academic environments (Brown et al., 2018).

Martirosyan et al. (2015) cited language proficiency as a primary cause of concern for international students that adversely affects their academic performance inside and outside the University classroom. English as an additional language is a recognised risk factor of poor academic performance outcomes in CALD students (Attrill et al., 2015). Within higher education in Australia, language barriers are a significant source of stress for overseas students who may experience difficulties in understanding Australian accents, feel challenged by domestic students’ use of Australian English vernacular, miss non-verbal nuances and have limited cognisance of accepted social norms (Jeong et al., 2011; O’Neill, 2011). In the health professional arena, studies of international nursing students in Australia reveal that fewer communication difficulties are reported when students are motivated to continually improve their language skills by incorporating local dialects and colloquialisms into their daily spoken and written language (Newton et al., 2016).

CALD students enrolled in health professional courses, which have medical and anatomical terminology embedded in them, may struggle to conform to the discipline-specific language requirements in their chosen subject (Velliaris & Breen, 2016). Strategic measures at the institutional level targeting English language proficiency and communication with fellow students and university staff have been shown to improve international students’ adaptation, resulting in higher self-esteem, better social and academic relationships, and enriched personal and educational learning (Contreras-Aguirre & Gonzalez, 2017). Enabling CALD students to build proficiencies in their use of the English language through support programs also represents a positive learning experience that facilitates the development of discipline-specific language and core professional attributes such as independence, role balance and client-centredness (Crawford & Candlin, 2013; Lim et al., 2016; O’Reilly & Milner, 2015).

#### Positive outlook

CALD students’ performance on measures of their ability to see the positive in people, situations and events, and persistence in pursuing goals despite obstacles and setbacks can be partially accounted for by cultural factors. In a sample dominated by first- and second-year students under the age of 24, all students irrespective of origin are likely to experience a period of academic and social adjustment within an unfamiliar setting. For CALD students transitioning from overseas, the effects of environmental factors may be heightened in the first 12 months of university education as a result of cultural shock, acculturative stress and feelings of disempowerment and isolation (Henning et al., 2012; Jeong et al., 2011; Wang et al., 2015).

Mikkonen et al.’s (2016) systematic review of the experiences of CALD nursing students’ learning in classroom and placement environments determined that promoting a positive learning environment minimises the challenges experienced by international students. This process may involve intercultural learning and understanding between students and supervisors, having daily debriefs with a supportive supervisor to share and solve problems with, alongside tutor–student interactions characterised by approachability, encouragement of open communications, the provision of regular feedback and adoption of transparent, consistent expectations (Attrill et al., 2015; Malau-Aduli, 2011; Rodger et al., 2011). Research findings from the health arena indicate that initiatives that instil proactive, open and positive attitudes in international students assist in minimising acculturative stress and the demands and challenges associated with cultural issues, leading to students assuming a sense of control in adapting to and having an appreciation for their academic learning environments (Gu et al., 2010; Mikkonen et al., 2016).

In the context of health professions education, engagement in group work and seeking clarification from colleagues, peers, clients and their families are important components. The creation of culturally sensitive programs that bolster CALD students’ self-esteem and positive outlook facilitates them to overcome educational and social barriers and ease their cultural transition as they embark on their professional education and training in host countries (Lim et al., 2016). The development of intercultural training that enhances educators’ understanding of west–east cultures and pedagogies removes prejudice against CALD students and reduces challenges imposed on CALD students by the education system (Grant et al., 2014; Wang et al., 2015). The aim of health professional education courses should be to enable high levels of emotional competence that is culturally sensitive and responsive in all students, irrespective of point of origin, that facilitate an in-depth understanding of their relevance in multi-disciplinary fields such as occupational therapy. The promotion of self-confidence and motivation in students’ academic and social endeavours is consistent with the OECD’s recent call for education policies and practices that help CALD students reach their academic potential, and increase their social integration, emotional adjustment and motivation to achieve (OECD, 2018). Thus, consideration needs to be given to new pedagogy development, such as social and educational activities that provide positive learning experiences, to support CALD students in building confidence in their ability to contribute and make an impact on the profession and their future clients.

### Social competencies

Investigation of students’ scores on key measures of relationship management and social awareness yielded particularly useful results in establishing significant differences at the *p* < 0.001 level in CALD and non-CALD student scores on the ESCI-U subscales of Empathy, Teamwork and Organisational Awareness. These findings are interesting because they present areas of concern for educators and it is useful to consider the cultural and linguistic factors that impact students’ performance in these domains.

#### Empathy

Empathic competencies comprise empathic skills such as communication and relational abilities which are based on mutual trust. Within this, the capacity to communicate verbally and non-verbally is used to control, clarify, support, understand, reconstruct and reflect on the patient’s thoughts and feelings and establish an interactive reciprocal empathic relationship in which long-term trust is developed (McMillan, 2010). The scores for non-CALD and CALD students in this domain indicate encouraging and respectable levels of empathy, particularly in a sample dominated by students in their early years of university education.

There is evidence to suggest that students’ empathic skills increase consistently and substantially as they progress through year levels of undergraduate health professional programs (McKenna et al., 2011). The scores on the Empathy subscale are consistent with the vocational nature of the health professions which typically attract students who have a desire to deploy their skills and knowledge to assist patients and clients in need of clinical and therapeutic intervention (Wood, 2016).

The significant difference observed in students' scores on the Empathy subscale may reflect environmental factors that heighten acculturative stress in CALD students. For example, research of international nursing and pharmacy students in Australia report challenges experienced in relation to spoken language, the negotiation of professional roles and expectations that make it difficult for CALD students to develop a rapport with academic staff, domestic students, clinical supervisors and clients (Crawford & Candlin, 2013; Gilligan & Outram, 2012). There is evidence however that CALD health science students who experience positive interactions in university classroom and practice education settings increase their empathy levels by building their intercultural sensitivity and awareness of cultural diversity (Green et al., 2008; Myhre, 2011). This emphasises the relevance of and need for culturally sensitive curricula designed to provide experiences and initiatives that strengthen empathy levels in international and domestic students.

Research indicating higher levels of empathic competence in female-dominant health professions such as occupational therapy is congruent with the composition of the sample in the current study (Ferri et al., 2017; Foster et al., 2017). Allied with evidence that empathy increases throughout course progression, the identification of female students’ higher capacity for learning empathic competencies offers encouragement to course directors of health professional programs with a high proportion of female CALD students. It suggests that when aligned with the adjustment of learning approaches that take into account cultural differences—for example, by educating academic and clinical staff on cultural and linguistic variables—students can be supported to improve their learning and implementation of empathic skills in practice (Mikkonen et al., 2016).

It has also been demonstrated that international students undertaking practice placements during the third and fourth years of occupational therapy programs become more familiar with typical Australian customs, leisure activities and environments (Gu et al., 2010) This results in students developing greater sensitivity and social awareness, thus facilitating their understanding of providing empathic and holistic services and improved interactions and connectivity with clients (Lalor et al., 2019).

#### Teamwork

The ability to work with others towards a shared goal, actively participate, share responsibilities and rewards and contribute to the capability of the team are essential attributes of health professionals. Being able to read a group’s emotional currents, understand power relationships and identify influencers, networks and dynamics are also core requirements. Non-CALD students’ performance on these measures of social competence represents an encouraging finding, as it suggests the demonstration of open dispositions, engagement, a willingness to share feelings and the empowerment of core values that facilitate students’ emerging professional identity (Brown et al., 2019).

It also highlights the role of interaction management skills (the skills to start, continue, and end a conversation smoothly) in occupational therapy students which are significantly associated with desirable professional behaviours such as competent communication skills (Yu et al., 2019). Although CALD students recorded respectable scores in this dimension, the regression analysis results indicate a significant difference in their performance compared to their non-CALD peers, suggesting the impact of cultural and linguistic factors on international students’ teamwork and organisational interaction capabilities in the western education context.

Previous studies of CALD students within Australia and internationally have highlighted the role of the acculturation process in determining the extent to which CALD students succeed academically and socially (Attrill et al., 2015; Lillyman & Bennett, 2014). According to Wang et al. (2015), when making decisions in a group context CALD students’ cultural requirement to respect others’ opinions may override the need to express their own thoughts, while their tendency for cooperation could be interpreted by academic staff in host countries as dependence. A proposed solution to overcome this issue is a third party with knowledge about both cultures who acts as facilitator to interpret perceptual differences and upskill both CALD students and educators in understanding the potential behavioural and attitudinal differences (Wang et al., 2015). Teaching and learning within the university setting that places international students in active roles, offers safe social learning environments and actively encourages community and peer interactions which have been shown to benefit CALD students’ knowledge construction and social integration (Lillyman & Bennett, 2014).

There is evidence that English language competency is important for CALD students (Jeong et al., 2011; Seibold et al., 2007) in making them feel part of the team (Myhre, 2011). In their investigation of Chinese students enrolled at Australian universities, Wang et al. (2015) reported that engagement with locals improved students’ English and communication skills, as well as strengthened their ability to make friends and develop a sense of belonging. To enrol in a university course in Australia, CALD students from overseas need to meet the English language requirements, such as the International English Test System (IELTS) score ranging from 5.5 to 7 depending on the course students are applying for admission to. Some students also need to achieve the English requirements for professional registration upon graduation (e.g. an overall IELTS score of 7 for health professional registrations in Australia). This means the expectations of English language skills are more explicit to CALD students and educators, compared to the implicit requirements of social-relational skills that are linked to language, communication and interaction with others.

The authors recommend universities provide opportunities for social engagement as an initiative to improve CALD students’ overall social and learning experiences. Australia being a multicultural country, occupational therapists in Australia need to be equipped with abilities to work with colleagues and patients from multicultural backgrounds. CALD students in turn also provide invaluable intercultural learning opportunities for non-CALD students to develop cultural competencies (Yu et al., 2017) that are imperative for health practice. These approaches are shown to provide students with insights into their own emotional and social competencies which are integral to the promotion of self-awareness, an improved ability to work with fellow team members and the building of supportive bonds with colleagues and clients (Gavriel, 2015). Within the health care disciplines, promoting the development of CALD students’ emotional and social intelligence competencies has been proven to generate positive effects on team cohesion and students’ interpersonal skills (Brown et al., 2016).

#### Organisational awareness

For students enrolled in health professional programs, it is essential that they learn to navigate organisational structures, beginning with the university setting in the early years of study, as well as within practice education contexts as they undertake clinical placements. The navigation process is stressful for all students, but the unique set of cultural and linguistic challenges experienced by CALD students represents a substantial barrier in adapting to and thriving within new and unfamiliar environments.

The findings from the regression analysis infer that familiarity with systems and processes in the academic setting facilitates higher coping thresholds in non-CALD students and easier social movement inside and outside the university classroom. While the lecture theatre or tutorial room may represent a relatively ‘safe’ environment for CALD students, the wider social context and work settings when undertaking fieldwork constitute challenging and difficult environments for students who may lack proficiency in written and spoken English (Edgecombe et al., 2013; Gilligan & Outram, 2012; Lalor et al., 2019; O’Reilly & Milner, 2015). This can lead to feelings of isolation and alienation often compounded by stress from perceived peer competition and the unrealistic expectations of, and criticism from, academic and/or practice staff and patients (Bertram Gallant et al., 2015). This serves to illustrate the importance of intercultural communication competence training within health sciences curricula that focusses on reducing communication barriers. Evidence suggests that such initiatives succeed in generating positive intergroup attitudes towards CALD students and intercultural encounters in which the protagonists feel less threatened (Mak et al., 2014).

Further initiatives from the nursing and medical arenas target CALD students’ interactions with fellow students, academic staff and supervisors. These include peer mentoring programs and homestay periods to develop socially supportive local networks; workshops to facilitate students’ increased participation in hands-on practical activities; and opportunities for informal interaction with academic staff and domestic students as a means for CALD students to improve their conversational skills (Abu-Arab & Parry, 2015; Henning et al., 2012). Where communication and expectations are open and transparent, acculturative stress is reduced and successful CALD inter-student and academic staff relationships are built from the outset (Attrill et al., 2015). This assists CALD students to develop their social resources to manage and develop further their emotional and social intelligence.

Both groups of students recorded their lowest scores on the social competencies subscales that measured conflict management, influencing, leadership, coaching and mentoring. Regression analysis confirmed there were statistically significant differences in CALD and non-CALD students’ performance across these measures. These findings reinforce the potential impact of cultural factors on students’ capabilities when managing emotionally tense situations, resolving conflicts, striving to have a positive impact on others and utilising persuasive skills to gain others’ support. This raises important considerations for educators concerning supporting students both in the university classroom and on professional practice placements where these skills would be required.

Students transitioning from Confucian-based education systems can struggle to adapt to the requirements of Western pedagogy in which students are expected to apply critical thinking, be active participants in classroom discussions and become independent learners (Lim et al., 2016). Educational remedial initiatives such as offering longer periods for positive reflection, group work activities, role play scenarios and early supportive orientation for entry into academic and social settings at university have been shown to equip, motivate and empower CALD students with the necessary skills and attributes to assist and inform them in overcoming cultural and linguistic barriers to their professional development (Grant & McKenna, 2003; Jeong et al., 2011; Myhre, 2011). Providing CALD and non-CALD students with positive learning experiences will facilitate them to translate their learning into professional capacities and develop their social attitudinal and behavioural competencies within an authentic, supportive and welcoming learning environment in the university setting.

## Limitations and future research

This study drew on a convenience sample of occupational therapy students from a single education university program. Therefore, caution is needed when generalising the findings beyond these students. With data generated using a self-report questionnaire design, the potential for bias self-reporting and CALD students’ cultural bias of their understanding towards the questionnaire items must also be acknowledged.

Further investigation in this area is recommended with participants recruited from multiple occupational therapy programs in Australia. It is also recommended to generate additional insights that add to the knowledge base on the emotional and social intelligence competencies of CALD students enrolled in broader health programs in Australia and internationally. Specifically, qualitative studies that generate narrative data about students’ coping mechanisms and longitudinal research that tracks changes in students’ emotional and social competencies over course progression may identify other factors that impact the development of emotional and social capacities in culturally and linguistically diverse students.

## Conclusion

This study adds to the growing body of evidence of the challenges faced by international students, and more specifically extends the findings by comparing emotional and social intelligence in English-speaking domestic and CALD students. The findings demonstrate that CALD and non-CALD students differ significantly in all emotional and social competencies deemed imperative from Western perspectives, most notably in the areas of social awareness and relationship management. CALD students can be disadvantaged by these differences in emotional and social competencies impacting their educational performance, practice education experiences, and interpersonal and social relationships with peers and educators. The accommodation of students’ cultural and linguistic diversities in relation to emotional and social competencies also represents a challenge for academic and fieldwork educators. The results observed in the current research highlight the need to promote intercultural education and develop culturally sensitive higher education curricula and university as well as practice environments with broader international perspectives.

Full social integration of CALD students into university life, promotion of their acquisition of effective emotional competencies, as well as intercultural understanding among all students, academic educators, and practice educators should be encouraged through proactive and meaningful initiatives. Curriculum should introduce elements (such as cultural awareness and sensitivity workshops) that assist non-CALD students to explore their own social and emotional skills, and develop social and emotional skills essential to manage university life, social expectations and preferred learning styles in the western context. Learning and social activities (e.g. cultural interviews, scenario discussions and reflections, and simulation) that provide practice opportunities as well as enhance mutual understanding, interaction, and integration between CALD and non-CALD students and/or educators are recommended. These activities need to be incorporated into and scaffolded throughout curriculum. In strengthening the emotional and social capacities of CALD students, their positive learning experiences will ensure that international education is meaningful, valued, and sustainable. Further, the constructive contributions these students make across the health care professions at national and international levels will be augmented.

## Data Availability

Not applicable.

## References

[CR1] Abu-Arab A, Parry A (2015). Supervising culturally and linguistically diverse (CALD) nursing students: A challenge for clinical educators. Nurse Education in Practice.

[CR2] Arthur N (2017). Supporting international students through strengthening their social resources. Studies in Higher Education.

[CR3] Attrill S, McAllister S, Lincoln M (2015). Predictors of professional placement outcome: Cultural background, English speaking and international student status. Perspectives in Medical Education.

[CR4] Australia Government Department of Education. (2019). *International student data monthly summary. Research document.* Australia Government Department of Education. https://internationaleducation.gov.au/research/International-Student-Data/Documents/MONTHLY%20SUMMARIES/2019/Aug%202019%20MonthlyInfographic.pdf.

[CR5] Banjong DN (2015). International students’ enhanced academic performance: Effects of campus resources. Journal of International Students.

[CR6] Beagan BL (2015). Approaches to culture and diversity: A critical synthesis of occupational therapy literature. Canadian Journal of Occupational Therapy.

[CR7] Beagan BL, Chacala A (2012). Culture and diversity among occupational therapists in Ireland: When the therapist is the ‘diverse’ one. British Journal of Occupational Therapy.

[CR8] Bertram Gallant T, Binkin N, Donohue M (2015). Students at risk for being reported for cheating. Journal of Academic Ethics.

[CR9] Bickel G, Jenson D (2012). Understanding cultural dissonance to enhance higher education academic success. International Scholarly and Scientific Research & Innovation.

[CR10] Boyatzis RE (2016). Commentary on Ackley (2016): Updates on the ESCI as the behavioural level of emotional intelligence. Consulting Psychology Journal: Practice and Research.

[CR11] Boyraz G, Legros DN, Berger WB (2020). Self-criticism, self-compassion, and perceived health: Moderating effect of ethnicity. The Journal of General Psychology.

[CR12] Brown T, Bourke-Taylor H, Isbel S, Gustaffsson L, McKinstry C, Logan A (2018). Exploring similarities and differences among the self-reported academic integrity of Australian occupational therapy domestic and international students. Nurse Education Today.

[CR13] Brown T, Williams B, Etherington J (2016). Emotional intelligence and personality traits as predictors of occupational therapy students’ practice education performance: A cross-sectional study. Occupational Therapy International.

[CR14] Brown T, Yu M-L, Hewitt A, Isbel ST, Bevitt T, Etherington J (2019). Exploring the relationship between resilience and practice education placement success in occupational therapy students. Australian Occupational Therapy Journal.

[CR15] Chang J (2015). The interplay between collectivism and social support processes among Asian and Latino American college students. Asian American Journal of Psychology.

[CR16] Contreras-Aguirre HC, Gonzalez EGY (2017). Experiences of international female students in US graduate programs. College Student Journal.

[CR17] Crawford T, Candlin S (2013). A literature review of the language needs of nursing students who have English as a second/other language and the effectiveness of English language support programmes. Nurse Education in Practice.

[CR18] Di Lorenzo R, Venturelli G, Spiga G, Ferri P (2019). Emotional intelligence, empathy and alexithymia: A cross-sectional survey on emotional competence in a group of nursing students. Acta Biomed for Health Professions.

[CR19] Duffin, E. (2020). *Top host destination of international students worldwide 2019*. Report document. https://www.statista.com/statistics/297132/top-host-destination-of-international-students-worldwide/#statisticContainer.

[CR20] Edgecombe K, Jennings M, Bowden M (2013). International nursing students and what impacts their clinical learning: Literature review. Nurse Education Today.

[CR21] Ferri P, Rovesti S, Panzera N, Marcheselli L, Bari A, Di Lorenzo R (2017). Empathic attitudes among nursing students: A preliminary study. Acta Bio-Medica: Atenei Parmensis.

[CR22] Foster K, Fethney J, McKenzie H, Fisher M, Harkness E, Kozlowski D (2017). Emotional intelligence increases over time: A longitudinal study of Australian pre-registration nursing students. Nurse Education Today.

[CR23] Gavriel J (2015). The self-directed learner in medical education.

[CR24] Gilligan C, Outram S (2012). Culturally and linguistically diverse students in health professional programs: An exploration of concerns and needs. Education Health.

[CR25] God YT, Hongzhi H (2019). Intercultural challenges, intracultural practices: How Chinese and Australian students understand and experience intercultural communication at an Australian university. Higher Education.

[CR26] Grant, L., & Strong, J., Xu, X., Popp, P., & Sun, Y. (2014). *West meets east: Best practices from expert teachers in the U. S. and China.* Alexandria.

[CR27] Grant E, McKenna L (2003). International clinical placements for undergraduate students. Journal of Clinical Nursing.

[CR28] Green BF, Johansson I, Rosser M, Tengnah C, Segrott J (2008). Studying abroad: A multiple case study of nursing students’ international experiences. Nurse Education Today.

[CR29] Greenland, S., Bhatia, B., Saleem, M. A., & Misra, R. (2021 September 09). Australia can rebound to be international students’ destination of choice when borders reopen. *The Conversation*. https://theconversation.com/australia-can-rebound-to-be-international-students-destination-of-choice-when-borders-reopen-167347.

[CR30] Group of Eight Australia. (2021). *Go8 submission to the Australian Strategy for International Education 2021–2030*. https://go8.edu.au/go8-submission-to-the-australian-strategy-for-international-education-2021-2030.

[CR31] Gu Q, Schweisfurth M, Day C (2010). Learning and growing in a ‘foreign’ context: Intercultural experiences of international students. Compare: A Journal of Comparative and International Education.

[CR32] Hart CS, Sriprakash A (2018). Understanding cultural dissonance and the development of social identities. International Studies in Sociology of Education.

[CR33] Hecht ML, Chin Y-J, Durlak JA, Domitrovich CE, Weissberg RP, Gullotta TP (2015). Culture and social and emotional competencies. Handbook of social and emotional learning: Research and practice.

[CR34] Henning MA, Krägeloh C, Moir F, Doherty I, Hawken SJ (2012). Quality of life: International and domestic students studying medicine in New Zealand. Perspectives in Medical Education.

[CR35] Institution of International Education (IIE). (2019). *Number of international students in the United States hits all-time high.* Report document.https://www.iie.org/Why-IIE/Announcements/2019/11/Number-of-International-Students-in-the-United-States-Hits-All-Time-High#:~:text=The%20total%20number%20of%20international,total%20U.S.%20higher%20education%20population.

[CR36] International Education Advisory Council (IEAC). (2013). *Australia – Educating globally*. Report document. Commonwealth of Australia. https://internationaleducation.gov.au/International-network/Australia/InternationalStrategy/theCouncilsReport/Documents/Australia%20%E2%80%93%20Educating%20Globally%20FINAL%20REPORT.pdf.

[CR37] Jain R (2019). Inclusive pedagogy: Tapping cognitive dissonance experienced by international students. Writing and Pedagogy.

[CR38] Jeong SY, Hickey N, Levett-Jones T, Pitt V, Hoffman K, Norton CA, Ohr SO (2011). Understanding and enhancing the learning experiences of culturally and linguistically diverse nursing students in an Australian bachelor of nursing program. Nurse Education Today.

[CR39] Korn Ferry. (2017). *Emotional and social competency inventory: Research guide and technical manual*. Korn Ferry.

[CR40] Lalor L, Yu M-L, Brown T, Thyer L (2019). Occupational therapy international undergraduate students’ perspectives on the purpose of practice education and what contributes to successful practice learning experiences. British Journal of Occupational Therapy.

[CR41] Lee D-C, Haines T, Maneephong, s., & Zeng, Q. (2019). Barriers to fieldwork placements for international higher degree students: A systematic literature review. Australian Journal of Career Development.

[CR42] Li H (2017). The ‘secrets’ of Chinese students’ academic success: Academic resilience among students from highly competitive academic environments. Educational Psychology.

[CR43] Li Z, Heath MA, Jackson AP, Allen GE, Fischer L, Chan P (2017). Acculturation experiences of Chinese international students who attend American universities. Professional Psychology: Research and Practice.

[CR44] Lillyman S, Bennett C (2014). Providing a positive learning experience for international students studying at UK universities: A literature review. Journal of Research in International Education.

[CR45] Lim JW, Honey A, Du Toit S, Chen Y-W, Mackenzie L (2016). Experiences of international students from Asian backgrounds studying occupational therapy in Australia. Australian Occupational Therapy Journal.

[CR46] Mak AS, Brown PM, Wadey D (2014). Contact and attitudes toward international students in Australia: Intergroup anxiety and intercultural communication emotions as mediators. Journal of Cross-Cultural Psychology.

[CR47] Malau-Aduli B (2011). Exploring the experiences and coping strategies of international medical students. BMC Medical Education.

[CR48] Malau-Aduli BS, Ross S, Adu MD (2019). Perceptions of intercultural competence and institutional intercultural inclusiveness among first year medical students: A 4-year study. BMC Medical Education.

[CR49] Martirosyan NM, Hwang E, Wanjohi R (2015). Impact of English proficiency on academic performance of international students. Journal of International Students.

[CR50] McKenna L, Boyle M, Brown T, Williams B, Molloy A, Lewis B, Molloy L (2011). Levels of empathy in undergraduate midwifery students: An Australian cross-sectional study. Women and Birth.

[CR51] McMillan LR (2010). Teaching nursing students empathic communication: A mandate from the Code of Ethics for Nursing. Online Journal of Health Ethics.

[CR52] Mikkonen K, Elo S, Kuivila H-M, Tuomikoski A-M, Kääriäinen M (2016). Culturally and linguistically diverse healthcare students’ experiences of learning in a clinical environment: A systematic review of qualitative studies. International Journal of Nursing Studies.

[CR53] Min MC, Islam M, N., Wang, L., & Takai, J. (2018). Cross-cultural comparison of university students’ emotional competence in Asia. Current Psychology.

[CR54] Mitchell C, Fabbro L, Shaw J (2017). The acculturation, language and learning experiences of international nursing students: Implications for nursing education. Nurse Education Today.

[CR55] Mitchell L, Paras A (2018). When difference creates dissonance: Understanding the ‘engine’ of intercultural learning in study abroad. Intercultural Education.

[CR56] Myhre K (2011). Exchange students crossing language boundaries in clinical nursing practice. International Nursing Review.

[CR57] Newton L, Pront L, Giles TM (2016). Experiences of registered nurses who supervise international nursing students in the clinical and classroom setting: An integrative literature review. Journal of Clinical Nursing.

[CR58] O’Neill F (2011). From language classroom to clinical context: The role of language and culture in communication for nurses using English as a second language: A thematic analysis. International Journal of Nursing Studies.

[CR59] O’Reilly SL, Milner J (2015). Supporting culturally and linguistically diverse students during clinical placement: strategies from both sides of the table. BMC Medical Education.

[CR60] Organization for Economic Cooperation and Development (2018). The resilience of students with an immigrant background: Factors that shape well-being.

[CR61] Rai D (2017). Assessment of emotional intelligence and emotional maturity of undergraduate students. International Journal of Humanities and Social Sciences.

[CR62] Richards CA, Doorenbos AZ (2016). Intercultural competency development of health professions students during study abroad in India. Journal of Nursing Education and Practice.

[CR63] Rodger S, Fitzgerald C, Davila W, Millar F, Allison H (2011). What makes a quality occupational therapy practice placement? Students’ and practice educators’ perspectives. Australian Occupational Therapy Journal.

[CR64] Seibold C, Rolls C, Campbell M (2007). Nurses on the move: Evaluation of a program to assist international students undertaking an accelerated bachelor of nursing program. Contemporary Nurse.

[CR65] Talarico JF, Varon AJ, Banks SE, Berger JS, Pivalizza EG, Medina-Rivera G, Metro DG (2013). Emotional intelligence and the relationship to resident performance: A multi-institutional study. Journal of Clinical Anesthesia.

[CR66] The Government of United Kingdom. (2019). *International education strategy global potential, global growth.* Report document.https://assets.publishing.service.gov.uk/government/uploads/system/uploads/attachment_data/file/799349/International_Education_Strategy_Accessible.pdf.

[CR74] U.S. Department of Education. (2021). Joint statement of principles in support of international education, United States. https://educationusa.state.gov/sites/default/files/intl_ed_joint_statement.pdf

[CR67] Velliaris DM, Breen P (2016). An institutional three-stage framework: Elevating academic writing and integrity standards of international pathway students. Journal of International Students.

[CR68] Wang CC, Andre K, Greenwood KM (2015). Chinese students studying at Australian universities with specific reference to nursing students: A narrative literature review. Nurse Education Today.

[CR69] Wood DF (2016). Mens sana in corpore sano: Student well-being and the development of resilience. Medical Education.

[CR70] Yilmazoglu, Y. (2021). How universities can win back international students. *University World News: The Global Window for Higher Education*. https://www.universityworldnews.com/post.php?story=2021020814252452.

[CR71] Young JT (2017). Confucianism and accents: Understanding the plight of Asian international students in the U.S. Journal of International Students.

[CR72] Yu M-L, Brown T, Farnworth L (2017). Embracing international students in occupational therapy higher education in Australia: Challenge or asset?. Australian Occupational Therapy Journal.

[CR73] Yu M-L, Brown T, Thyer L (2019). The association between undergraduate occupational therapy students’ listening and interpersonal skills and performance on practice education placements. Scandinavian Journal of Occupational Therapy.

